# Simple and Accurate Border Detection Algorithm for Melanoma Computer Aided Diagnosis

**DOI:** 10.3390/diagnostics10060423

**Published:** 2020-06-22

**Authors:** Cataldo Guaragnella, Maria Rizzi

**Affiliations:** DEI—Department of Electrics and Information Engineering, Politecnico di Bari, 70125 Bari, Italy; cataldo.guaragnella@poliba.it

**Keywords:** automatic segmentation, edge detection, melanoma, skin lesion, computer aided detection and diagnosis, skin cancer

## Abstract

The interest of the scientific community for computer aided skin lesion analysis and characterization has been increased during the last years for the growing incidence of melanoma among cancerous pathologies. The detection of melanoma in its early stage is essential for prognosis improvement and for guaranteeing a high five-year relative survival rate of patients. The clinical diagnosis of skin lesions is challenging and not trivial since it depends on human vision and physician experience and expertise. Therefore, a computer method that makes an accurate extraction of important details of skin lesion image can assist dermatologists in cancer detection. In particular, the border detection is a critical computer vision issue owing to the wide range of lesion shapes, sizes, colours and skin texture types. In this paper, an automatic and effective pigmented skin lesion segmentation method in dermoscopic image is presented. The proposed procedure is adopted to extract a mask of the lesion region without the adoption of other signal processing procedures for image improvement. A quantitative experimental evaluation has been performed on a publicly available database. The achieved results show the method validity and its high robustness towards irregular boundaries, smooth transition between lesion and skin, noise and artifact presence.

## 1. Overview

**Figure 1:**
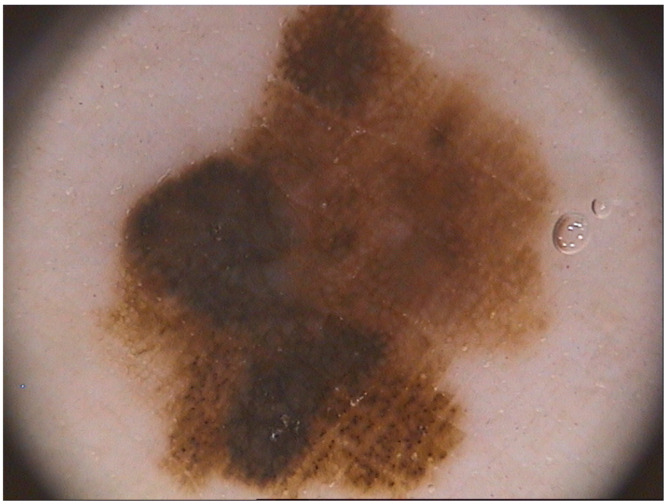
Image of a melanoma taken from a publicly available database. Melanoma is a serious form of skin cancer whose dangerousness is the consequence of its ability to spread to other organs more rapidly if not treated at an early stage. An early diagnosis usually increases the chances for successful treatments [5]. Because of the appearance similarities between benign and malign skin lesions, identification of skin lesion type by the naked eye could be difficult for even well-trained specialists. New approaches have been developed in the last decades for an early and accurate diagnosis of melanoma such as diagnostic algorithms (pattern analysis, ABCDE-rule, Menzies method, 7-point checklist, etc.) applied on images acquired by dermoscopic technology, a non-invasive imaging technique. It enables a magnified and clear visualization of skin morphological structures that are not discernible by an examination with the naked eye [6,7]. Researchers have indicated that dermoscopy enhances diagnostic results only if well-trained dermatologists adopt it. An inverse relation between the use of this image technique and its diagnostic effectiveness towards melanoma early detection is shown if physician is not adequately trained [8]. Therefore, the subjectivity of the analysis carried out by the specialist (which depends on human vision and dermatologist clinical experience) gives to the melanoma diagnosis the behaviour of hard reproducibility.

Computer aided diagnosis (CADx) of melanoma (Figure 1) makes an objective evaluation of skin lesions and provides reproducible diagnosis by eliminating the inter and intra-observer variabilities typical of the examination by specialist [1]. In this perspective, the aim of CADx systems is to increase clinician performance by helping in the early identification and localization of potential abnormalities [2]. Therefore CADx system operates as an automated second opinion or as a double reading system that supports dermatologists in early assessment of skin cancer and in the follow-up of pigmented skin lesions [3].

A key step in the development of CADx systems for the automatic diagnosis of skin lesions is the border detection/segmentation phase as it heavily influences the performance that the method can achieve. An accurate and precise segmentation and border detection highlight information which is relevant for a high reliable diagnosis such as the lesion area asymmetry and irregularity. Moreover, blue-white areas, atypical pigment networks and globules, which are some of the relevant clinical features for skin lesion classification, can be automatically identified in the presence of an accurate segmentation [4]. Unfortunately, low contrast between lesion and surrounding tissue, non-uniform lighting, border irregularity and indistinctness, hair and air/oil bubbles, variegated colours of skin areas are just some of the factors that make the segmentation phase a hard task.

In this paper a simple method for skin lesion segmentation which is able to perform an accurate border detection is proposed. The input of the procedure is the dermoscopic image-under-test while the output are the segmentation of the skin lesion and a detailed border identification.

## 2. Proposed Approach

**Figure 2:**
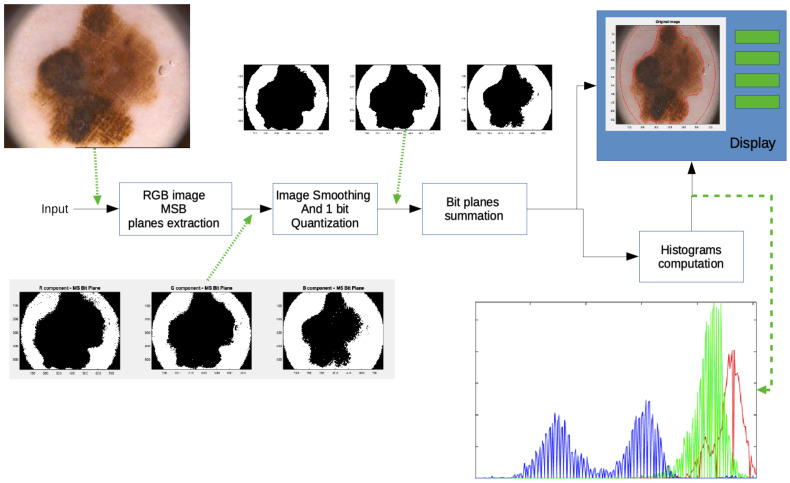
Block diagram of the conceived approach.

In Figure 2 the functional block diagram of the conceived method is sketched. In particular for each stage the input and the produced output are shown.

Because of each Red, Green and Blue (RGB) color image is quantized with 8 bits and it is represented in an unsigned integer format, the image under test can represented as a three-component vector described as follows:

$$\left[R\left(x,y\right),G\left(x,y\right),B\left(x,y\right)\right]=\left[\sum_{i=0}^{7}r_{i}\left(x,y\right)\cdot 2^{i},\sum_{i=0}^{7}g_{i}\left(x,y\right)\cdot 2^{i},\sum_{i=0}^{7}b_{i}\left(x,y\right)\cdot 2^{i}\right]$$

where ($r_{i}$, $g_{i}$, $b_{i}$) are the bit values of the generic pixel having (x, y) coordinates.

In the first stage the RGB image is split in its three color components, Red (R) Green (G) and Blue (B) and a subsequent bit plane splitting procedure is applied. As the human eye is mostly sensible to the higher values of the luminance and luminance difference, only the Most Significant Bit Plane is retained for the three R, G, B color components. The three bit planes, thus form three color component bidimensional binary images expressed as follows:

$$\left[R_{MSB}\left(x,y\right),G_{MSB}\left(x,y\right),B_{MSB}\left(x,y\right)\right]=\left[r_{7}\left(x,y\right),g_{7}\left(x,y\right),b_{7}\left(x,y\right)\right]$$

where the subscript “MSB” stands for Most Significant Bit.

To avoid the presence of spurious pixels in the obtained binary components and to get a binary image as output, a low pass filtering procedure followed by a one bit quantization are implemented in the second stage. In particular, a 2D Hanning shaped filter of 7 × 7 dimension has been used.

The filtered images are, then, summed up for the segmentation achievement. Because of the values arising from the sum are between zero (the image portion outside the inspection area) to three (presence of one in all the MSB planes of RGB images), a 4 level segmentation is obtained.

The same procedure could also be applied to the other bit planes both for giving a more complete description of the whole lesion area and for highlighting the different regions composing the pigmented lesion, at detriment of the computational load. In the implemented procedure, the adoption of only the most significant bit planes makes possible the achieving of an accurate image (lower number of regions) to be proposed to the specialist for the classification of the segmented areas and the assessing of suspicious lesions. Since the proposed algorithm has been conceived as a tool to be integrated into a CADx system, the method enables specialists to select the segmented areas of major interest for a subsequent diagnosis. The other details, that could be achieved by processing the remaining bit planes, might be usefully take into account in the CADx system to propose a possible lesion scoring to the physician.

In addition to the lesion border detection, the conceived procedure provides the physician with the color histograms of each segmented region. Information contained in every histogram, which concerns the distribution of colors in the detected areas, could help dermatologist during the phase of lesion diagnosis.

The output of the implemented system represents the input for a next Diagnosis Support System able to classify the segmented area as melanoma or no-melanoma by implementing one of the well-known diagnostics algorithms such as the ABCDE rule.

The developed procedure is an efficient approach with respect to the required computational load since the well known approaches to facilitate border identification, such as masking, image resizing, filtering, color space conversions, hair removal, reduction of noise and artifacts are not implemented.

## 3. Results

For the performance evaluation of the implemented system, the publicly available PH2 database is adopted as test-bench which contains 200 skin lesion dermoscopic images (80 atypical nevi, 80 common nevi and 40 melanoma) [9]. The performance of the implemented procedure is assessed by comparing the method outputs with the ground truths indicated by expert clinicians contained in the database.

The commonly used metrics for the check and validation of proposed algorithms in image segmentation are defined in the scientific literature. In this paper the accuracy is evaluated which is defined as the observed agreement between the procedure results and the physician opinion about the lesion under test. It is defined as the ratio between the sum of true detected pixels, both for the case of positive and negative areas, and the number of examined pixels. Stated TP(true positive) the number of lesion pixels correctly classified as lesion inside the image under test, TN (true negative) the number of non-lesion pixels correctly identified as non-lesion, FP (false positive) the number of non-lesion pixels incorrectly identified as lesion and FN (false negative) the number of lesion pixels incorrectly identified as non-lesion, the Accuracy is expressed as follows:

$$Accuracy=\frac{TP+TN}{TP+FP+TN+FN}$$

Adopting the ground-truths provided by the PH2 database, an average accuracy of about 95.6% is achieved.

## 4. Discussion and Future Development

**Figure 3:**
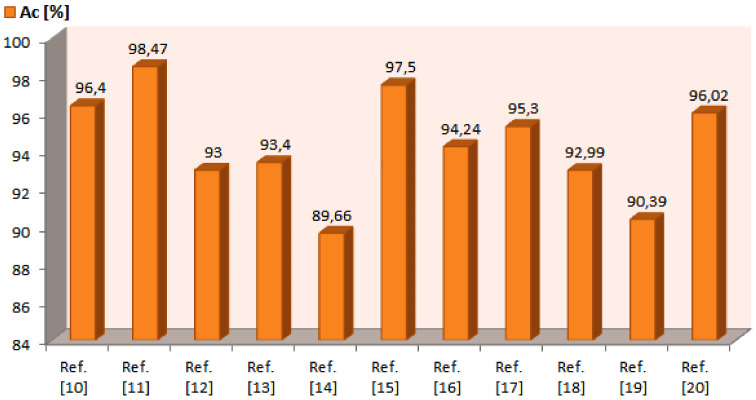
Though several scientific papers deal with this research issue, new studies may be suggested both to overcome the problems still outstanding and to achieve high performance results. To make a reliable and meaningful benchmark with different border detection methods indicated in literature, the same database should be adopted as test-bench of the procedures to be compared. It is well known that database characteristics influence the achieved performance of a CAD method and, therefore, the same procedure could produce different results when different data sets are used. Some of the most recent and popular algorithms which use the PH2 data set have been analyzed [10,11,12,13,14,15,16,17,18,19,20]. Despite the simplicity of the proposed approach, the comparative assessment reported in the figure show that the results achieved by the proposed method are positioned in the high end of the performance indicated in the scientific literature.

**Figure 4:**
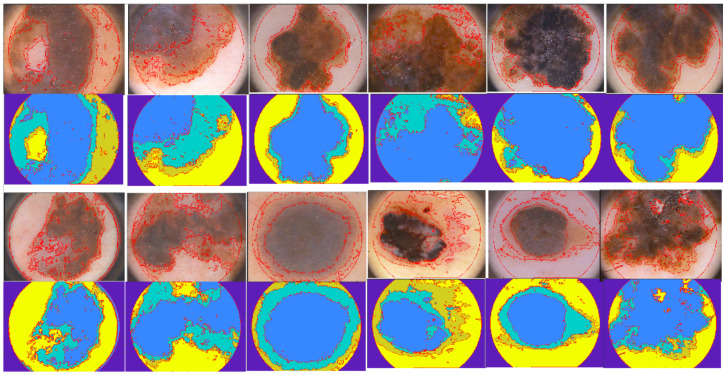
Some of the experimental results obtained by the procedure are reported. In particular, original images of melanoma, which are shown with over-imposed contours obtained in the segmentation phase, are compared with the segmented areas obtained as outputs of the proposed method. In the performance evaluation of the conceived procedure, all the data-set images have been tested, contrary to what some other papers indicated in literature have performed [4]. The reason of this rejection is based on the image characteristics. In fact, several images of the PH2 database are heavily affected by poor illumination and presence of hair, some others contain multiple lesions and others are connected to the boundary edge of the provided image. Therefore, the method achieved the above indicated results taking also in consideration these challenging dermoscopic images. Independently of the performance achieved by the computer method, it is to emphasize that the effect of border detection imprecision on a CADx system can be only assessed when the segmentation procedure is integrated in a diagnostic system.

Even though our method exhibits performance comparable with the results (Figure 3) of the most popular and recent algorithm indicated in literature, it shows many distinctive characteristics, which prove the performance especially in terms of signal processing, tool applicability and application conditions. In fact, the implemented method shows that a careful use of image processing techniques which also adopt Boolean operators, makes it possible to reach performance in line with the other methods without the implementation of a pre-processing phase (aimed to reduce noise, to improve image quality and to remove hair) and the recourse to complex detection systems (based on neural networks, heuristic or probabilistic approaches. Some of the segmentation obtained for melanoma section of the PH2 data set are reported in Figure 4. Therefore the following benefits characterize the implemented method:

- it is an open architecture which can be upgraded individually to improve the border detection procedure;
- it is positioned in the high end of the performance indicated in literature;
- it is an approach that needs no training and subsequent validation for the test procedure;
- it is an efficient approach with respect to the required computational load;
- it is designed to be sensitive even with images characterized by irregular borders

Future developments will test the implemented procedure with other public available databases so to evaluate the performance stability regards to the image variability present into a data-set (i.e., in some database the region of interest takes into account the lesion and its proximity, others contain the single lesion or part of the lesion crossing its margin).

## References

[1] Rizzi M., D’Aloia M. (2014). Computer aided system for breast cancer diagnosis. Biomed. Eng. Appl. Basis Commun..

[2] Rizzi M., D’Aloia M., Cice G. (2015). Computer aided evaluation (CAE) of morphologic changes in pigmented skin lesions. Lect. Notes Comput. Sci..

[3] Messadi M., Bessaid A. (2012). Segmentation and characterization of skin tumors images used for aided diagnosis of melanoma. J. Biomed. Sci..

[4] Rizzi M., Guaragnella C. (2020). Skin Lesion Segmentation Using Image Bit-Plane Multilayer Approach. Appl. Sci..

[5] ”Melanoma: Incidenza e Mortalità”. https://www.infomedics.it/therapeutic-areas/melanoma/epidemiologia.html.

[6] Carrera C., Marchetti M.A., Dusza S.W., Argenziano G., Braun R.P., Halpern A.C., Jaimes N., Kittler H.J., Malvehy J., Menzies S.W. (2016). Validity and Reliability of Dermoscopic Criteria Used to Differentiate Nevi From Melanoma A Web-Based International Dermoscopy Society Study. JAMA Dermatol..

[7] Celebi M.E., Codella N., Halpern A. (2019). Dermoscopy Image Analysis: Overview and Future Directions. IEEE J. Biomed. Health Inform..

[8] Ho L. (2019). Fully automated GrowCut-based segmentation of melanoma in dermoscopic images. J. Young Investig..

[9] Mendonca T., Ferreira P.M., Marques J.S., Marcal A.R.S., Rozeira J. PH2-A dermoscopic image database for research and benchmarking. Proceedings of the 2013 35th Annual International Conference of the IEEE Engineering in Medicine and Biology Society (EMBC).

[10] Vesal S., Malakarjun Patil S., Ravikumar N., Maier A.K., Stoyanov D., Taylor Z., Sarikaya D., McLeod J., Ballester M.A.G., Codella N.C.F., Martel A., Maier-Hein L., Malpani A., Zenati M.A. (2018). A Multi-task Framework for Skin Lesion Detection and Segmentation. OR 2.0 Context-Aware Operating Theaters, Computer Assisted Robotic Endoscopy, Clinical Image-Based Procedures, and Skin Image Analysis.

[11] Olugbara O.O., Taiwo T.B., Heukelman D. (2018). Segmentation of Melanoma Skin Lesion Using Perceptual Color Difference Saliency with Morphological Analysis. Math. Probl. Eng..

[12] Peng Y., Wang N., Wang Y., Wang M. (2019). Segmentation of dermoscopy image using adversarial networks. Multimed. Tools Appl..

[13] Pathana S., Gopalakrishna Prabhub K., Siddalingaswamya P.C. (2018). Hair detection and lesion segmentation in dermoscopic images using domain knowledge. Med. Biol. Eng. Comput..

[14] Pennisi A., Bloisi D., Nardi D., Giampetruzzi A.R., Mondino C., Facchiano A. (2016). Skin lesion image segmentation using Delaunay Triangulation for melanoma detection. Comput. Med. Imaging Graph..

[15] Khan M.A., Akram T., Sharif M., Shahzad A., Aurangzeb K., Alhussein M., Haider S., Altamrah A. (2018). An implementation of normal distribution based segmentation and entropy controlled features selection for skin lesion detection and classification. BMC Cancer.

[16] Bi L., Kim J., Ahn E., Kumar A., Fulham M., Feng D. (2017). Dermoscopic Image Segmentation via Multistage Fully Convolutional Networks. IEEE Trans. Biomed. Eng..

[17] Bi L., Kim J., Ahn E., Kumar A., Feng D., Fulham M. (2019). Step-wise integration of deep class-specific learning for dermoscopic image segmentation. Pattern Recognit..

[18] Ünver H.M., Ayan E. (2019). Skin Lesion Segmentation in Dermoscopic Images with Combination of YOLO and GrabCut Algorithm. Diagnostics.

[19] Patino D., Avendano J., Branc J.W., Frangi A., Schnabel J., Davatzikos C., Alberola-López C., Fichtinger G. (2018). Automatic Skin Lesion Segmentation on Dermoscopic Images by the Means of Superpixel Merging. Medical Image Computing and Computer Assisted Intervention—MICCAI 2018.

[20] Aljanabi M., Özok Y.E., Rahebi J., Abdullah A.S. (2018). Skin Lesion Segmentation Method for Dermoscopy Images Using Artificial Bee Colony Algorithm. Symmetry.

